# Comment on “A randomized double-blind trial of intranasal dexmedetomidine versus intranasal esketamine for procedural sedation and analgesia in young children”

**DOI:** 10.1186/s13049-025-01403-5

**Published:** 2025-05-13

**Authors:** Jiajing Wang

**Affiliations:** 1 Department of Anesthesiology, Nanfang Hospital, Southern Medical University, The key Laboratory of Precision Anesthesia & perioperative Organ Protection, Guangzhou, 510515 Guangdong China; 2 Department of Anesthesiology, Zengcheng Campus Nanfang Hospital, Southern Medical University, Guangzhou, 511338 Guangdong China

**Keywords:** Dexmedetomidine, Esketamine, Children, Sample size, Sedation

## Abstract

This comment critiques a trial comparing intranasal dexmedetomidine (DEX) and esketamine (sKET) for pediatric procedural sedation. Despite a large effect size, the small sample (*n* = 29) likely caused false-negative results (*p* = 0.09), necessitating larger trials. Safety concerns (e.g., aspiration risk), unaddressed long-term psychological outcomes, and limited pharmacokinetic data (delayed DEX onset, prolonged duration) challenge clinical applicability. Future studies should integrate objective measures and long-term follow-up.

Dear Editors,

We read with great interest the article published in the Scandinavian Journal of Trauma, Resuscitation and Emergency Medicine entitled “A randomized double-blind trial of intranasal dexmedetomidine versus intranasal esketamine for procedural sedation and analgesia in young children” [1]. This article is the first to compare the efficacy and safety of intranasal dexmedetomidine (DEX) with that of intranasal esketamine (sKET) for procedural sedation and analgesia in young children aged 1 to 3 years in the emergency department setting. The innovation of DEX lies in the convenience and safety of intranasal administration, and it is the first time to verify the clinical application potential of DEX in young children with trauma. While the study provides valuable insights, we would like to highlight a few potential limitations:

Firstly, in terms of sample size and statistical power, even if the authors had observed a larger effect size (d = 1.37), the results did not reach significance (*p* = 0.09) because of the insufficient sample size, suggesting that the findings may be “false negative”. For example, the study by Poonai et al. [2] showed that a large sample size not only improves statistical power but also provides clear guidance for clinical practice through accurate dose-response analyses. In contrast, the original study failed to reach statistical significance (*p* = 0.09) even though a large effect size was observed (a difference of 4 points in the FLACC) due to insufficient sample size (only 30 cases), highlighting the key effect of sample size on the reliability of the results. Future studies are recommended to expand the sample size and multicenter design to improve the statistical power of the analysis and the generalitability of the conclusions.

Secondly, in the interpretation of the results, the observed effect size (Hedges’g = 1.33) was a very large effect according to Cohen’s criteria (g = 0.2 for small effect, 0.5 for medium effect, and 0.8 for large effect), indicating a significant difference in analgesic efficacy between intranasal DEX and sKET. However, due to insufficient sample size (*n* = 29), the study failed to reach statistical significance (*p* = 0.09), suggesting a “false negative risk “(type II error) [3].

Thirdly, in terms of how to balance the depth of sedation and safety, the plausibility of a Ramsay score of 3 (responsive to sleep and eyebrow tapping) in the emergency department setting: whether it increases the risk of aspiration, and whether additional monitoring is needed. Cote et al. [4], in their guidelines for monitoring and managing sedation in pediatric patients, describe the indications and risks of deep sedation in children, especially those who are not fasting. In addition, the authors’ safety data are limited. Although there were no serious adverse events, a small sample size may underestimate low-probability risks [2] (e.g., respiratory depression).

Fourthly, there are limitations in terms of parental satisfaction and children’s experience. The evaluation of parents’ satisfaction may be affected by confounding factors such as the depth of sedation of children. For example, deeper sedation (such as Ramsay score ≥ 3) may indirectly improve parents’ subjective evaluation of treatment by reducing children’s crying or physical activity, rather than directly reflecting analgesic effect or drug safety. Future studies need to incorporate multi-dimensional measures (e.g., video behavioral analysis, physiological parameter monitoring) to distinguish the independent effects of sedation and analgesia. Furthermore, long-term psychological outcomes such as children’s postoperative anxiety or pain memory were not tracked. Noel et al. [5] and Weisman et al. [6] have shown that children’s memory of medical pain can significantly influence their adherence to future treatments and anxiety levels. The lack of such data in this study limits judgments about the long-term value of the drug. Future studies are recommended to include long-term follow-up of children’s behavior (e.g., the degree of cooperation in the next medical visit).

Fifthly, there is a lack of pharmacokinetic data. The onset of action of intranasal DEX is 15 to 40 min [2, 7], and the timing of administration needs to be planned in advance. It is suitable for non-urgent but moderate sedation procedures in the emergency department. However, for rapid onset or very short-term operation, combination with other drugs (such as ketamine) or intravenous administration is recommended. Its long half-life of DEX, with a duration of action of 1 to 2 h [2, 7], may affect the length of stay in the recovery room and the efficiency of emergency department turnover.

Additionally, the parent questionnaire was subject to subjective bias. It is suggested that the combination of objective data sources (such as independent scores after video recording of pain behavior, physiological indicators) and traditional methods can significantly reduce subjective bias and improve the scientific validity and reliability of research results [8].

In conclusion, we appreciate that the authors provide a new evidence-based basis for procedural sedation and analgesia in young children. We believe these considerations may help to further refine sedation protocols and stimulate valuable scientific discourse in this important area of perioperative medicine. We look forward to continued progress in this area.

## Data Availability

No datasets were generated or analysed during the current study.

## References

[1] Nikula A, Lundeberg S, Ryd Rinder M, Lääperi M, Sandholm K, Castrén M, Kurland L. A randomized double-blind trial of intranasal Dexmedetomidine versus intranasal Esketamine for procedural sedation and analgesia in young children. Scand J Trauma Resusc Emerg Med. 2024;32(1):16. 10.1186/s13049-024-01190-5. PMID: 38439043; PMCID: PMC10913425. 38439043 10.1186/s13049-024-01190-5 PMC10913425

[2] Poonai N, Sabhaney V, Ali S, Stevens H, Bhatt M, Trottier ED, Brahmbhatt S, Coriolano K, Chapman A, Evans N, Mace C, Creene C, Meulendyks S, Heath A. Optimal Dose of Intranasal Dexmedetomidine for Laceration Repair in Children: A Phase II Dose-Ranging Study. Ann Emerg Med. 2023;82(2):179–190. 10.1016/j.annemergmed.2023.01.023. Epub 2023 Mar 3. PMID: 36870890. 10.1016/j.annemergmed.2023.01.023 36870890

[3] Pugh SL, Torres-Saavedra PA. Fundamental statistical concepts in clinical trials and diagnostic testing. J Nucl Med. 2021;62(6):757–64. 10.2967/jnumed.120.245654. Epub 2021 Feb 19. PMID: 33608427; PMCID: PMC8729862. 33608427 10.2967/jnumed.120.245654 PMC8729862

[4] Coté CJ, Wilson S, AMERICAN ACADEMY OF PEDIATRICS; AMERICAN ACADEMY OF PEDIATRIC DENTISTRY. Guidelines for Monitoring and Management of Pediatric Patients Before, During, and After Sedation for Diagnostic and Therapeutic Procedures. Pediatrics. 2019;143(6):e20191000. 10.1542/peds.2019-1000. PMID: 31138666. 10.1542/peds.2019-1000 31138666

[5] Noel M, Chambers CT, McGrath PJ, Klein RM, Stewart SH. The influence of children’s pain memories on subsequent pain experience. Pain. 2012;153(8):1563–1572. 10.1016/j.pain.2012.02.020. Epub 2012 May 3. PMID: 22560288. 10.1016/j.pain.2012.02.020 22560288

[6] Weisman SJ, Bernstein B, Schechter NL. Consequences of inadequate analgesia during painful procedures in children. Arch Pediatr Adolesc Med. 1998;152(2):147-9. 10.1001/archpedi.152.2.147. PMID: 9491040. 10.1001/archpedi.152.2.147 9491040

[7] Miller JW, Balyan R, Dong M, Mahmoud M, Lam JE, Pratap JN, Paquin JR, Li BL, Spaeth JP, Vinks A, Loepke AW. Does intranasal Dexmedetomidine provide adequate plasma concentrations for sedation in children: a Pharmacokinetic study. Br J Anaesth. 2018;120(5):1056–65. 10.1016/j.bja.2018.01.035. Epub 2018 Mar 13. PMID: 29661383. 29661383 10.1016/j.bja.2018.01.035

[8] Palinkas LA, Mendon SJ, Hamilton AB. Innovations in mixed methods evaluations. Annu Rev Public Health. 2019;40:423–42. 10.1146/annurev-publhealth-040218-044215. Epub 2019 Jan 11. PMID: 30633710; PMCID: PMC6501787. 30633710 10.1146/annurev-publhealth-040218-044215 PMC6501787

